# A new genus and species of Dictyopharidae (Homoptera) from Rovno and Baltic amber based on nymphs

**DOI:** 10.3897/zookeys.130.1775

**Published:** 2011-09-24

**Authors:** Alexander F. Emeljanov, Dmitry E. Shcherbakov

**Affiliations:** 1 Zoological Institute, Russian Academy of Sciences, Universitetskaya Emb. 1, St. Petersburg, 199034 Russia; 2 Borissiak Paleontological Institute, Russian Academy of Sciences, Profsoyuznaya St. 123, Moscow, 117997 Russia

**Keywords:** Fulgoroidea, planthoppers, Dictyopharinae, Orthopagini, Achilidae, fossil, Baltic amber, Eocene, morphology, wax plates

## Abstract

*Alicodoxa rasnitsyni* **gen. et sp. n.** (Dictyopharinae: Orthopagini) is described based on a nymph from Rovno amber; it also occurs in Baltic amber. A small additional wax plate dorsal to the large wax plate of abdominal tergites VI–VIII is first reported in this and other genera of Dictyopharidae. A lectotype is designated for *Pseudophana reticulata* Germar & Berendt, 1856 transferred to *Protepiptera* (Achilidae): *Protepiptera reticulata* (Germar & Berendt, 1856), **comb. n.**

## Introduction

The family Dictyopharidae is poorly represented in the fossil record. Its oldest member, described from the Upper Cretaceous (Santonian) of Taimyr, has been assigned to the extinct tribe Netutelini (Emeljanov 1983). Several Cenozoic genera have been attributed to the family, but most of them need re-examination to determine their systematic position in more detail (see Szwedo et al. 2004, Szwedo 2008). Recently, Szwedo (2008) described a new monotypic genus from Baltic amber and placed it in a tribe of its own, Worskaitini, which he considered closely related to Netutelini.

Two nymphs from Late Eocene Baltic amber were described by Germar and Berendt (1856) as *Pseudophana reticulata*, i.e. as Dictyopharidae in the modern sense(*Dictyophara* Germar = *Pseudophana* Burmeister). Based on the original illustrations, these two specimens have been reinterpreted by Emeljanov (1983), the 3rd instar as resembling Tropiduchidae, and the late instar (“pupa”) as belonging to Achilidae, most probably to the extant genus *Cixidia* Fieber. The late instar nymph (designated below as the lectotype of *Pseudophana reticulata*) indeed seems to be an achilid and presumably belongs to the extinct genus *Protepiptera* Usinger, which is similar to *Cixidia*, whereas the 3rd instar nymph is a dictyopharid. A photograph of yet another 3rd instar nymph of Dictyopharidae from Baltic amber has been published by Weitschat and Wichard (1998: pl. 46a). These two dictyopharid nymphs are conspecific with an excellently preserved nymph from Rovno amber, described below as a new genus and species and placed with substantial certainty into the extant tribe Orthopagini.

The Rovno amber from NW Ukraine is roughly contemporaneous to Baltic amber, both Late Eocene in age and containing similar but distinct insect faunas with many species in common (Perkovsky et al. 2010). The second planthopper species shared between the two ambers is *Protepiptera kaweckii* Usinger, 1939 (Achilini s.l.), the commonest Baltic amber achilid (redescribed by Emeljanov and Shcherbakov 2009). Other planthopper families recorded from Rovno amber are Tropiduchidae (Perkovsky and Bodgasarov 2009) and Cixiidae.

## Material and methods

The specimen was collected in Klesov, Rovno Region, Ukraine, and deposited in the amber collection of the Schmalhausen Institute of Zoology, National Academy of Sciencesof Ukraine, Kiev (SIZK). Morphological terminology follows (Emeljanov (1993, 1994). Photographs were taken with Leica MZ9.5 stereomicroscope and Leica DFC420 camera and post-processed using Helicon Focus software.

## Taxonomy

### Superfamily Fulgoroidea Latreille, 1807. Family Achilidae Stål, 1866. Tribe Achilini Stål, 1866, s.l. Genus Protepiptera Usinger, 1939

#### Protepiptera reticulata

(Germar & Berendt, 1856) comb. n.

http://species-id.net/wiki/Protepiptera_reticulata

Pseudophana reticulata Germar & Berendt, 1856: 16, pl. II, fig. 4b (pars)
Dictyophara reticulata (Germar & Berendt, 1856): Metcalf and Wade 1966: 126
Cixidia reticulata (Germar & Berendt, 1856): Szwedo et al. 2004: 85

##### Lectotype.

 Late instar nymph (“pupa”) in Baltic amber, former East Prussia (Germar and Berendt 1856: 16, pl. II, fig. 4b), designated here.

##### Remarks.

 Of the two syntype nymphs, the late instar is selected as a lectotype. The 3rd instar nymph, belonging to Dictyopharinae, is discussed under the new genus and species below. According to Szwedo et al. (2004), the type material was probably lost during World War II.

This species is possibly a senior synonym of *Pseudophana kaweckii* Usinger, 1939 based on adults and very common in Baltic amber. Other possible senior synonyms of *Pseudophana kaweckii* are *“Cixius” longirostris* Germar et Berendt, 1856 and *“Oliarus” oligocenus* Cockerell, 1910 (Emeljanov and Shcherbakov 2009).

### Family Dictyopharidae Spinola, 1839. Subfamily Dictyopharinae Spinola, 1839. Tribe Orthopagini Emeljanov, 1983

#### Genus Alicodoxa gen. n.

urn:lsid:zoobank.org:act:C730EB51-0600-45A1-8E17-993E07139A32

http://species-id.net/wiki/Alicodoxa

##### Type species.

*Alicodoxa rasnitsyni* sp. n.

##### Etymology.

 The genus and the type species are named in honour of our friend and colleague Prof. Alexandr Rasnitsyn. The grammatical gender is feminine.

##### Diagnosis.

 Metope not visible in dorsal aspect. Coryphe 1/3 longer than pronotal disc along midline. Pronotum deeply angulately emarginate posteriorly. Lateral carinae of mesonotal disc anteriorly converging at acute angle. Fore femur without subapical tooth. Abdominal tergites IV–V with 1–2 sensory pits displaced forwards from the row of pits. Tergites VI–VII with 2 medial and 2–3 lateral pits. Tergites VI–VIII with large lower and small upper wax plates, upper plate of tergite VII subdivided.

##### Remarks.

Similar to the extant genera *Orthopagus* Uhler and *Saigona* Matsumura, but in the nymphs of these latter the metope is visible from above, coryphe longer relative to pronotal disc, pronotum less emarginate posteriorly, and tergites VI–VII with 4 medial and 1–0 lateral pits. Subdivided upper wax plate of tergite VII is unknown in other Dictyopharidae. Other characters listed under Diagnosis assign the new genus to Orthopagini within Dictyopharinae (see Discussion).

#### Alicodoxa rasnitsyni sp. n.

urn:lsid:zoobank.org:act:F0581436-F029-46E3-9229-29D20AFF5140

http://species-id.net/wiki/Alicodoxa_rasnitsyni

Figs 1 5

##### Material.

 Holotype, 4th instar nymph, SIZK K-3719, Klesov, Rovno amber, Ukraine; Late Eocene. Syninclusions: Mycetophilidae, Sciaroidea, Symphypleona, Acari, stellate hairs. Petaloid blind fissures arising all around lateral margins of the nymph and directed nearly laterad completely separate its dorsal and ventral sides so that only parts of its mid and hind legs are visible from above. The ventral aspect is mostly masked with variously directed fissures and in some places with milky impurities.

##### Description.

 Nymph dark brown, ovoid, moderately elongate, 4.1 mm long, 2.2 mm wide; head projecting forwards beyond oval contour; dorsum finely transversely shagreened. Coryphe somewhat longer than wide; its lateral and anterior margins forming regular parabola; its posterior margin very shallowly concave, situated at about eye midlength in dorsal aspect. All carinae of coryphe, including posterior one and medial one, well developed. Metope (only partly visible) with medial areas somewhat widened at head apex; lateral areas with two rows of sensory pits, with an additional row of four smaller pits in between the two rows. Rostrum reaching beyond hind coxae, apical segment shorter than subapical one.

Pronotal disc strongly projecting forwards (2/3 of its median length situated anterior to level of posterior eye margins), its anterior margin truncate, almost straight, anterolateral angles rounded, lateral margins moderately diverging backwards. Posterior margin of pronotum with deep right-angled emargination reaching almost 1/3 of pronotal disc length. Pronotal disc slightly narrower and 1/4 shorter along midline than coryphe, bordered with distinct carinae along all free margins, medial carina also distinct, but posterolateral carinae undeveloped, and boundary between disc and lateral lobes traceable only as flexure of surface plane (posterior ends of these flexures close to points where lateral carinae of mesonotal disc approach pronotal margin). Sensory pits of pronotal disc and paradiscal areas forming one entity: disc with row of 4 large pits along lateral margin and 3 smaller pits in second, more medial row; paradiscal area with 6 large pits in marginal row (indistinctly subdivided into two groups, each with 3 pits) and 8–10 smaller pits in second row. Humeral area with 3 pits in main row and 1 additional pit; pectoral group of pits (about 7 in two tangled rows) situated as is usual in the family near posterior margin of paranotal lobe. Lateral and collateral carinae distinct. Mesonotal disc arrow-shaped anteriorly (its lateral carinae anteriorly converging at nearly acute angle, running parallel to posterior pronotal margin). Group of 6–7 pits (3 medial pits larger) situated laterad of lateral carinae. Fore wing pads mediad of subcostal carina with 3 pits (in triangle) in middle part and 1 pit near apex, and in costal area with 2 pits in middle part (against group of 3 pits). Posterior margins of fore and hind wing pads subparallel, directed obliquely posterolaterad. Metanotal disc rectangular, about 1.3 times as wide as long, with all carinae distinct. Medial group of pits laterad of disc similar to that on mesonotum; hind wing pad subapically with 2 pits in oblique row subparallel to posterior pad margin. Fore legs slightly widened, as long as mid legs. Fore femora without subapical tooth on posteroventral carina. Fore tibiae slightly flattened, lanceolate, widest about midlength. Mid tibiae not widened, relatively slender. Hind tibiae with 5 lateral spines including knee spine; apical teeth not possible to count. Hind tarsus three-segmented.

**Figures 1–3.**
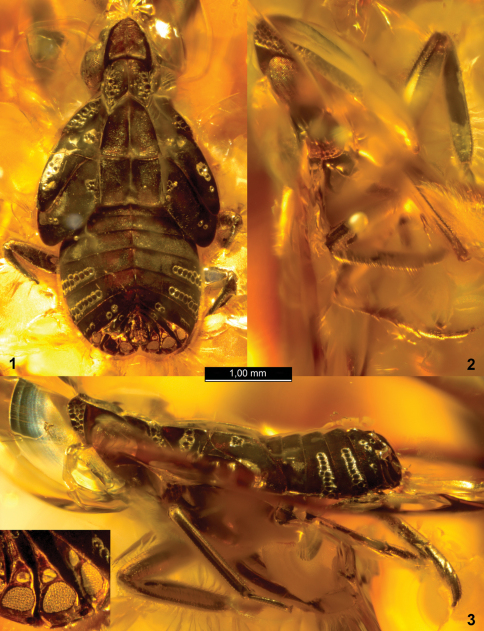
*Alicodoxa rasnitsyni* gen. et sp. n. (Orthopagini), 4th instar nymph, Rovno amber: **1** dorsal view **2** anteroventral view **3** lateral view (all to same scale); inset, wax plates of the right side, enlarged.

Abdomen with well developed middorsal carina, indistinct intermediate carinae on tergites IV–V, without sublateral carinae. Tergites I–III without pits; tergites IV–V with long (complete) rows of 8–10 pits, 5th or 6th pit (from body midline) displaced anteriad (sometimes there are 2 such pits, forming rudimentary second row anteriorly – see Fig. 1). Lateral areas of tergites IV–V with 3–4 pits in row or group. Tergites VI–VIII with several pits displaced medially (2 pits on VI, 2 pits on VII, 0–1 pit on VIII – absent at one side) and several pits laterally (ventrally: 3 pits on VI, 2 pits on VII, 1 pit on VIII). Lateral area with 3 pits on tergite VI, 2 pits on tergite VII, and 1 pit on tergite VIII. Tergite IX ventrally with pair of pits (1 pit at each side).

Wax plates situated in subtriangular posterolateral areas of tergites VI–VIII, separated from rest of tergite by carina and facing posteriad. Wax plates of uniform structure: large rounded lower (lateral) plate with discernible circular wax gland pores and small adjacent upper (medial) plate. Small upper wax plate on tergite VII subdivided, crossed by narrow chitinous bridge nearly perpendicular to body sagittal plane.

##### Remarks.

The three-segmented hind tarsus in the holotype of *Alicodoxa rasnitsyni* sp. n. indicates the 4th or 5th instar, while the fore wing pads not nearly reaching the apices of hind wing pads suggest the 4th instar (or 5th instar of a brachypterous planthopper, but the latter is not consistent with the presence of well developed abdominal wax plates).

In the nymphs of Dictyopharinae posterolateral carinae of the pronotal disc are usually absent, as in the new genus, and sensory pits of the second row are arranged more or less evenly across the imaginary boundary between pronotal disc and paradiscal area, though their number is not the same at the left and right sides.

**Figures 4–8.**
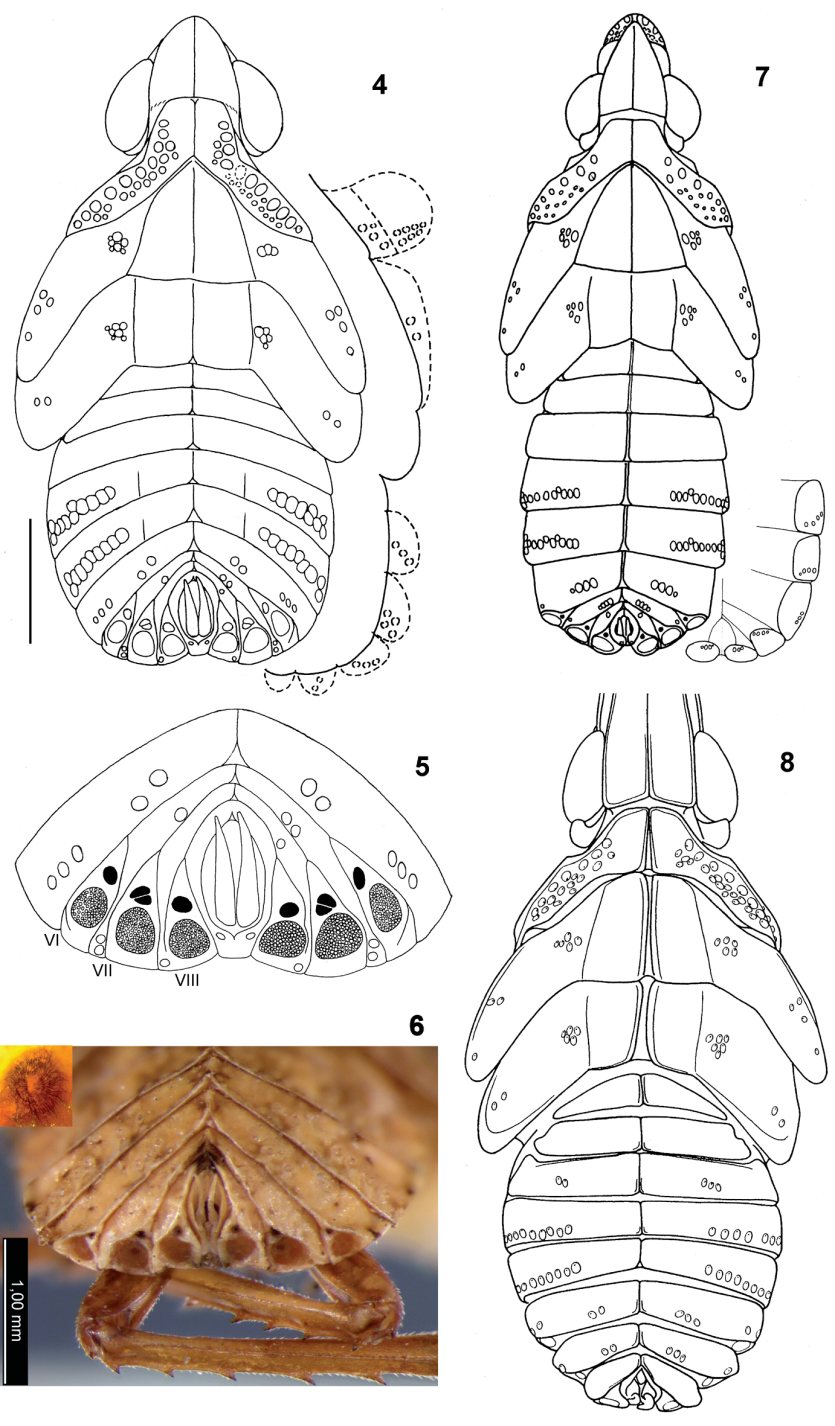
**4–5** *Alicodoxa rasnitsyni* gen. et sp. n., 4th instar nymph, Rovno amber: **4** dorsal view, slightly corrected and schematized; on the right, arrangement of sensory pits on the lateral body parts facing ventrad (scale bar, 1 mm) **5** posterior part of the abdomen, arrangement of wax plates and sensory pits; **6–7** *Saigona ussuriensis* (Lethierry) (Orthopagini), nymphs, recent: **6** 5th instar, posterior part of the abdomen with wax plates; inset, upper wax plate of segment VIII, enlarged **7** 4th instar, dorsal view; on the right, arrangement of sensory pits on ventrolateral parts of the tergites; **8** *Dictyophara pannonica* (Germar), 4th instar nymph, recent, dorsal view (apical part of the head not shown; after Emeljanov 1994).

The 3rd instar nymph from Baltic amber illustrated by Weitschat and Wichard (1998: pl. 46a) agrees with the holotype of *Alicodoxa rasnitsyni* sp. n. in all diagnostic characters. The original drawing of the 3rd instar nymph of „*Pseudophana reticulata”* (Germar and Berendt 1856: pl. II, fig. 4a) is inaccurate, e.g. the legs are shown too short and the sensory pits on the fore wing pad too numerous. Nevertheless, several salient features allow recognizing it as conspecific with the holotype of the new species. Therefore, *Alicodoxa rasnitsyni* sp. n. is recorded from both Baltic and Rovno amber.

## Discussion

*Alicodoxa* gen. n. is a typical member of Dictyopharinae in its general habitus and basic structural features. The subfamily assignment is confirmed by the presence of typical wax plates on the VI–VIII abdominal tergites (wax plates are undeveloped in another subfamily, Orgeriinae).

Nymphs of Dictyopharinae are rather uniform and difficult to identify, except for some aberrant forms. Nymphal characters to diagnose all the tribes have not been revealed yet, but there is a certain amount of morphological descriptions of varying accuracy and credibility (Wilson and McPherson 1981, Emeljanov 1993, 1994, Yang and Yeh 1994, McPherson and Wilson 1995). Several figures of nymphs in earlier papers (e.g. Sirrine and Fulton 1914, Logvinenko 1971) are not informative enough.

The descriptions scattered in the literature include those of nymphs (mainly of the last, 5th instar) from the tribes Dictyopharini, Orthopagini, Nersiini, Taosini, Phylloscelini, and Scoloptini. With the exception of Phylloscelini (genus *Phylloscelis* Germar), we examined nymphs from all these tribes plus the nymphs (yet undescribed) of the tribe Aluntiini, available in the collection of the Zoological Institute RAS. The nymphs of Lappidini, Capenini, Cleotychini, and Hastini remain unknown.

The wax plates of planthopper nymphs are originally confined to the abdominal tergites VI–VIII. The presence of 5 primary wax plates at each side of the tergites VI–VIII is characteristic of all Fulgoroidea nymphs except the primitive family Cixiidae (Emeljanov 2009). In many families these plates are greatly modified and variously reduced, but in some Fulgoridae, Nogodinidae, Ricaniidae, Flatidae, and Hypochthonellidae a complete set of wax plates is retained on at least one segment.

Within Dictyopharidae (Dictyopharinae) the structure of wax plates is quite uniform, except for the wax plates of segment VI often being small (*Dictyophara multireticulata* Mulsant & Rey) or absent (*Nersia* Stål*, Scolops* Schaum), but apparently only in the late instars (traced through developmental stages in *Nersia*: Wilson and McPherson 1981). In the holotype nymph of *Alicodoxa rasnitsyni* sp. n. a second, small, upper (medial) wax plate was discovered for the first time above the large, lower (lateral) one that was described in many genera (Wilson and McPherson 1981, Emeljanov 1990, 1993, Yang and Yeh 1994, McPherson and Wilson 1995). Subsequent examination has revealed the presence of the upper wax plates in nearly all the studied extant 5th instar nymphs from various tribes of Dictyopharinae, especially large ones in the nymphs of *Saigona ussuriensis* (Lethierry) (Figs 6–7). In these latter, at high magnification the upper wax plates show numerous dark striae radiating from the pale centre (Fig. 6, inset), the fine structure very different from that of the lower wax plates covered with densely packed circular wax gland pores.

In *Zanna tenebrosa* (Fabricius) of the subfamily Zanninae, considered the least advanced in the family Fulgoridae (Emeljanov 1979, Urban and Cryan 2009), there is a complete set of 5 wax plates at each side of segments VI–VIII, and the lower (lateral) plates differ in their structure from the remaining, uniformly built ones. In other Fulgoridae studied there are only 2 wax plates and only on the segments VII–VIII (Wilson and Wheeler 1992, Yang and Yeh 1994; in the former paper, the “dark brown sclerite” dorsal to the wax plate is apparently the upper wax plate). The lower wax plate is apparently homologous in Fulgoridae and Dictyopharidae, whereas the upper wax plate could be homologized to the row of 4 upper wax plates reduced to a single plate. This suggestion is corroborated by the upper wax plate of segment VII being subdivided in *Alicodoxa rasnitsyni* sp. n.

The new genus is assigned to the tribe Orthopagini based on the arrow-shaped anterior convergence of the lateral carinae of the mesonotal disc, combined with one or two sensory pits being displaced forwards from rows on the abdominal tergites IV–V; the latter character is found only in Orthopagini, the former also in Nersiini. The subapical tooth at the fore femur is characteristic of most Orthopagini but absent in several species of *Centromeria* Stål and in the new genus.

The tribe Orthopagini is presently distributed mainly in the Oriental and eastern Palearctic regions, plus three or four genera in the tropical Africa. The tribe Nersiini is Neotropical by origin and is confined to the New World (with only one subendemic genus, *Rhynchomitra* Fennah, in the Nearctic). Only the tribe Dictyopharini occurs now in Europe; in the nymphs of this tribe lateral carinae of the mesonotal disc are straight, not curved mediad, terminating separately at the anterior segment margin, and several pits on the abdominal tergites are displaced posteriad (not anteriad) from the row (Fig. 8).

The nymphs of Worskaitini are unknown; the adult of *Worskaito stenexi* Szwedo from Baltic amber differs from *Alicodoxa* gen. n. by the narrower, more elongate head. Because elongation of the head in Dictyopharinae, if it occurs, proceeds gradually from instar to instar, the head proportions of the 4th instar suggest that the head of adult *Alicodoxa rasnitsyni* sp. n. must be short and broad.

## Supplementary Material

XML Treatment for Protepiptera reticulata

XML Treatment for Alicodoxa

XML Treatment for Alicodoxa rasnitsyni
